# The role of *Apolipoprotein E4* on cognitive impairment in Parkinson’s disease and Parkinsonisms

**DOI:** 10.3389/fnins.2025.1515374

**Published:** 2025-02-20

**Authors:** Angenelle Eve Rosal, Sarah L. Martin, Antonio P. Strafella

**Affiliations:** ^1^Brain Health Imaging Centre, Centre for Addiction and Mental Health, Toronto, ON, Canada; ^2^Institute of Medical Sciences, Temerty Faculty of Medicine, University of Toronto, Toronto, ON, Canada; ^3^Translation and Computational Neurosciences Unit (TCNU), Faculty of Health and Education, Manchester Metropolitan University, Manchester, United Kingdom; ^4^Edmond J. Safra Parkinson Disease Program, Neurology Division, Toronto Western Hospital and Krembil Brain Institute, University Health Network, University of Toronto, Toronto, ON, Canada

**Keywords:** *Apolipoprotein E4*, Parkinsonism, DLB, cognitive impairment, neuroimaging, Parkinson’s disease

## Abstract

Cognitive impairment is a prevalent non-motor symptom of Parkinson’s disease (PD), increasing the risk of dementia as the disease progresses. Despite its clinical significance, the etiology of cognitive impairment in PD remains unclear. *Apolipoprotein E4 (APOE4)*, a well-known genetic risk factor of Alzheimer’s disease, has been studied for its potential role in PD-related cognitive impairment. However, findings have been conflicting and thus inconclusive, highlighting a need to critically evaluate the current research. Several studies using neuroimaging modalities have explored the brains of individuals with PD and atypical parkinsonian disorders who have *APOE4*. Some of these studies have identified distinct neuropathological changes that have been previously reported to be associated with cognitive impairments in those with Parkinsonisms. Here, we review the role of *APOE4* on cognitive impairment in PD and atypical Parkinsonisms using neuroimaging evidence. We will examine how *APOE4* may contribute to pathological changes within the brain and its association with cognitive impairment.

## 1 Introduction

Parkinson’s disease (PD) is a progressive, neurodegenerative disorder classically characterized by its motor symptoms of resting tremor, muscle rigidity, bradykinesia, and postural instability (Aarsland et al., 2017). However, non-motor symptoms also play a role toward the disease’s burden, with some appearing before clinical diagnosis and others emerging decades after motor deficits (Kalia and Lang, 2015; Aarsland et al., 2017). Cognitive impairment is among the most prominent non-motor symptoms. Over 30% of individuals newly diagnosed with PD have cognitive impairment (PD-MCI) and around 40% with PD-MCI may develop dementia (PDD) within 5 years of their diagnosis (Santangelo et al., 2015; Pedersen et al., 2017; Monastero et al., 2018). Additionally, a multi-center study found that approximately 80% of their newly diagnosed patients with PD who survived had dementia at a 20 year follow up (Hely et al., 2008). Due to this prevalence, previous studies established several risk factors of cognitive impairment in PD, with age being a notable predictor (Aarsland et al., 2017, 2021). Older PD patients have a higher risk in experiencing cognitive impairment, with the incidence of PDD increasing as they age (Hely et al., 2008; Santangelo et al., 2015; Aarsland et al., 2017, 2021; Monastero et al., 2018). As a result, aging is considered a key predictive marker for the development of PDD (Aarsland et al., 2017, 2021). Other established risk factors of PD-related cognitive impairment include visual hallucinations, lower education levels, presence of depression, as well as motor symptom severity (Aarsland et al., 2017, 2021). Despite these findings however, the etiology of cognitive impairment in PD remains poorly understood and inadequately researched in comparison to motor symptoms (Chaudhuri et al., 2006; Marinus et al., 2018). Therefore, determining the factors that contribute to the manifestation of cognitive impairment in PD is of great interest.

Genetic factors may contribute to cognitive impairment in PD (Collins and Williams-Gray, 2016). At present, this includes *Apolipoprotein E4 (APOE4)*, a well-established gene polymorphism that increases risk for Alzheimer’s Disease (AD) and has been associated with cognitive impairments (Reitz and Mayeux, 2010; Collins and Williams-Gray, 2016; Fernández et al., 2021; Fernández-Calle et al., 2022). The relationship between *APOE4* and cognitive function in subjects with PD has been examined, however, studies have produced contradictory conclusions. While several studies have linked *APOE4* to cognitive impairment in PD individuals (Pankratz et al., 2006; Monsell et al., 2014; Samat et al., 2017; Tropea et al., 2018; Szwedo et al., 2022), others found no association (Ezquerra et al., 2008; Kurz et al., 2009; Williams-Gray et al., 2009; Federoff et al., 2012).

Interestingly, the association of *APOE4* has also been studied in Dementia with Lewy Bodies (DLB), an atypical parkinsonian disorder that shares significant overlapping characteristics with both AD and PDD (Tsuang et al., 2013; van der Lee et al., 2018; Shiner et al., 2021; Nasri et al., 2022; Bousiges et al., 2023; Carceles-Cordon et al., 2023). DLB is the second most common dementia followed by AD, exhibiting similar neuropathological and clinical features to both disorders (Donaghy and McKeith, 2014; Jellinger and Korczyn, 2018; Carceles-Cordon et al., 2023). Notably, DLB and PDD share key similarities, as both are characterized as Lewy body diseases (LBD) due to the presence of Lewy body pathology, primarily composed of alpha-synuclein proteins (Donaghy and McKeith, 2014; Jellinger and Korczyn, 2018; Carceles-Cordon et al., 2023). However, the distinction between the two is controversially dependent on the timing of cognitive impairment; DLB is diagnosed when cognitive impairment occurs before parkinsonian motor symptoms arise, and PDD is diagnosed when cognitive impairment occurs after (Donaghy and McKeith, 2014; Jellinger and Korczyn, 2018; Carceles-Cordon et al., 2023). Though some studies have also identified that there is an association between *APOE4* with DLB (Tsuang et al., 2013; van der Lee et al., 2018; Shiner et al., 2021; Nasri et al., 2022; Bousiges et al., 2023), the role of *APOE4* toward DLB pathogenesis and its associated cognitive impairment also still remains unclear. As a result, preclinical studies have investigated *APOE4*’s role on potential mechanisms that may be linked with this non-motor symptom in PD and DLB (Dickson et al., 2018; Davis et al., 2020; Zhao et al., 2020). However, these studies are currently still limited, leading to significant gaps in our understanding of *APOE4*-dependent mechanisms underlying cognitive impairment in these parkinsonian disorders.

Neuroimaging studies have revealed that cognitive impairment in PD and DLB is associated with distinct neuropathological changes in the brain (Galvin et al., 2011; Garcia-Garcia et al., 2012; Irwin et al., 2012; Gomperts et al., 2013; Noh et al., 2014; Peraza et al., 2014; Segura et al., 2014; Blanc et al., 2016; Xu et al., 2016; O’Callaghan and Lewis, 2017; van der Zande et al., 2018; Chung et al., 2019; Chabran et al., 2020; Kantarci et al., 2020; Liu et al., 2021). Yet, analysis of these changes and their association with *APOE4* in PD is inconsistent, and are limited (Apostolova et al., 2012; Gomperts et al., 2012, 2013; Beyer et al., 2013; Campbell et al., 2013; Nicoletti et al., 2016; Mashima et al., 2017; Rane et al., 2018; Sampedro et al., 2019). *APOE4* may serve as a potential genetic biomarker for a higher risk of cognitive impairment in parkinsonian individuals, and therefore may have a significant clinical impact to allow early identification of at-risk individuals. As such, this review aims to enhance clarity regarding the potential contribution of *APOE4* in cognitive impairment and the associated neuropathological changes in PD and atypical Parkinsonisms, specifically DLB, using human neuroimaging studies.

## 2 Apolipoprotein E4 (APOE4)

APOE4 is one of the 3 major isoforms (APOE2, APOE3, APOE4) of the *Apolipoprotein E (APOE)* gene in humans, which are encoded by the *E2, E3,* and *E4* alleles, respectively (Fernández-Calle et al., 2022). Considering *APOE* is polymorphic in humans, the three alleles form six major genotypes (*APOE2/2, APOE3/3, APOE4/4, APOE2/3, APOE2/4,* and *APOE3/4*). The *APOE* gene is then translated to the APOE protein, which is primarily produced by astrocytes, microglia, oligodendrocytes, pericytes, the choroid plexus, and neurons in the central nervous system (CNS) (Husain et al., 2021; Fernández-Calle et al., 2022). The APOE protein plays various functions in the brain, including synaptic plasticity, neural signaling, modulation of the immune response, neuronal repair, and lipid transport (Husain et al., 2021; Fernández-Calle et al., 2022).

An association between cognitive function and *APOE4* has been numerously reported in the literature. Several studies discovered that *APOE4* carriers, whether healthy or diagnosed with AD, demonstrate an accelerated rate of cognitive impairment after follow-up when compared to non-carriers (Cosentino et al., 2008; Rawle et al., 2018; Emrani et al., 2020; Gharbi-Meliani et al., 2021). These individuals also exhibited a greater decline in specific cognitive domains, including executive function and language, as well as have an increased dementia risk (Slooter et al., 1998; Rawle et al., 2018; Gharbi-Meliani et al., 2021). Therefore, *APOE4* is distinguished to be one of the several factors that contribute to cognitive impairment (Qian et al., 2017; Gharbi-Meliani et al., 2021).

*APOE4* has also been extensively studied for its association with AD, revealing that individuals carrying at least one *APOE4* allele have an increased risk of developing the disease (Liu et al., 2013; Fernández-Calle et al., 2022). Over the years, its role toward AD pathogenesis has been reviewed to involve amyloid-beta protein dependent mechanisms (Theendakara et al., 2018; Hunsberger et al., 2019; Yamazaki et al., 2019; Husain et al., 2021; Serrano-Pozo et al., 2021; Martens et al., 2022; Raulin et al., 2022; Pires and Rego, 2023). Notably, enhanced aggregations of amyloid-beta proteins, one of the key hallmarks of AD protein pathology, has been widely examined to be associated with cognitive impairment in AD research (Westerman et al., 2002; Zhu et al., 2017; Pang et al., 2022). In preclinical studies, amyloid-beta precursor (APP) transgenic mice, an established animal model for amyloid-beta deposition and AD, exhibit changes in cognitive domains greatly impaired in AD patients, including learning and memory dysfunction (Westerman et al., 2002; Jahn, 2013; Webster et al., 2013; Zhu et al., 2017; Pang et al., 2022). In a corresponding manner, AD patients positive for amyloid-beta proteins not only exhibit memory impairments, but also deficits in executive and visuospatial function, language, attention, and overall cognitive performance (Lim et al., 2014; Meng et al., 2019). Thus, these findings suggest a direct association between enhanced amyloid-beta proteins in AD pathogenesis that may adversely affect cognitive function. Remarkably, numerous studies proposed potential mechanisms by which *APOE4* may contribute to the aggregation and deposition of amyloid-beta proteins to understand why it is a major risk factor of AD. This includes its role in increasing amyloid-beta production and formation, such as accelerating the development of amyloid-beta fibrils and oligomers (Liu et al., 2017; Yamazaki et al., 2019; Serrano-Pozo et al., 2021; Pires and Rego, 2023; Zhang et al., 2023). For instance, APOE4 has been examined to accelerate early seeding of amyloid-beta pathology in mice models, resulting in a significant enhancement of amyloid-beta production and oligomerization (Liu et al., 2017). Inhibition of APOE4 using anti-sense oligonucleotides during the seeding stage of amyloid-beta proteins reduced this pathology (Huynh et al., 2017). Other studies found that *APOE4* may increase amyloid-beta synthesis by enhancing *APP* transcription (Huang et al., 2017; Theendakara et al., 2018). Further, impaired amyloid-beta clearance has been linked to *APOE4* as well (Castellano et al., 2011; Fitz et al., 2012; Verghese et al., 2013; Wildsmith et al., 2013; Theendakara et al., 2018; Hunsberger et al., 2019; Liu et al., 2019; Serrano-Pozo et al., 2021; Raulin et al., 2022; Pires and Rego, 2023). APOE4 has been shown to be less effective in modulating receptor-mediated clearance of amyloid-beta by disrupting the binding between clearance receptors with the proteins, likely due to APOE4’s competitive binding of these receptors on astrocytes and microglia (Verghese et al., 2013; Theendakara et al., 2018; Hunsberger et al., 2019; Yamazaki et al., 2019; Serrano-Pozo et al., 2021; Raulin et al., 2022; Pires and Rego, 2023). It also prevents proteolytic clearance of amyloid by blocking the activity of enzymes crucial for the process (Cook et al., 2003; Wildsmith et al., 2013).

*APOE4* has also been linked to AD risk, pathogenesis, and its associated cognitive impairment through mechanisms independent of amyloid-beta pathology. This includes enhancing the presence of neurofibrillary tangles, which is primarily composed of hyperphosphorylated tau protein and is one of the other hallmarks of AD protein pathology (Theendakara et al., 2018; Hunsberger et al., 2019; Husain et al., 2021; Raulin et al., 2022; Pires and Rego, 2023). Preclinical studies using AD mouse models have highlighted that hyperphosphorylated tau is associated with cognitive impairment (Rowe et al., 2007; Zhang et al., 2020; Chen and Yu, 2023). Notably, APOE4 has also been found to influence tau-related processes, including enhancing phosphorylation, deposition and aggregation when compared to APOE2 and APOE3, as observed using post-mortem brains and transgenic mice (Tesseur et al., 2000; Brecht et al., 2004; Andrews-Zwilling et al., 2010). Thus, it is possible that *APOE4* may play a role in exacerbating tau-related neurodegeneration to overall contribute to cognitive impairment in AD. This speculation is further supported by a study conducted by Emrani et al. (2020), reporting that AD *APOE4* carriers exhibit greater tau accumulation when compared to non-carriers, which was associated with greater memory impairments (Emrani et al., 2020). Another suggested mechanism of APOE4’s relation with AD pathogenesis is its association with lipid transport, especially involving cholesterol (Yamazaki et al., 2019; Husain et al., 2021). APOE serves as a major lipid transporter in the CNS by delivering cholesterol to neurons (Liu et al., 2013; Yamazaki et al., 2019; Husain et al., 2021). Notably, APOE4 has been associated with deficient cholesterol transport, as it is less effective in transporting brain cholesterol when compared to APOE3, as well as linked to cholesterol accumulation (Rapp et al., 2006; Husain et al., 2021; Piccarducci et al., 2023). Since cholesterol is crucial for synaptic formation, axonal growth, and neuronal health, all of which are essential for learning and memory, APOE4’s association with impaired cholesterol transport has been inferred to contribute to AD risk and its associated cognitive impairment as well (Liu et al., 2013). Other mechanisms thought to be associated with *APOE4* in AD are neuroinflammation, mitochondrial deregulation, synaptic deficits, reduced vascular integrity as well as impaired glucose metabolism and autophagy (Theendakara et al., 2018; Hunsberger et al., 2019; Yamazaki et al., 2019; Husain et al., 2021; Serrano-Pozo et al., 2021; Martens et al., 2022; Raulin et al., 2022; Pires and Rego, 2023). These processes are also associated with cognitive impairment as observed in AD patients and transgenic mice, exacerbating neurodegeneration (Dragicevic et al., 2010; Sharma et al., 2015; Kuang et al., 2019; Loera-Valencia et al., 2019; Nicastro et al., 2020; Barisano et al., 2022; Kumar et al., 2022; Lecca et al., 2022; Eisenmenger et al., 2023; Yin, 2023; del Campo et al., 2024).

**Figure 1:**
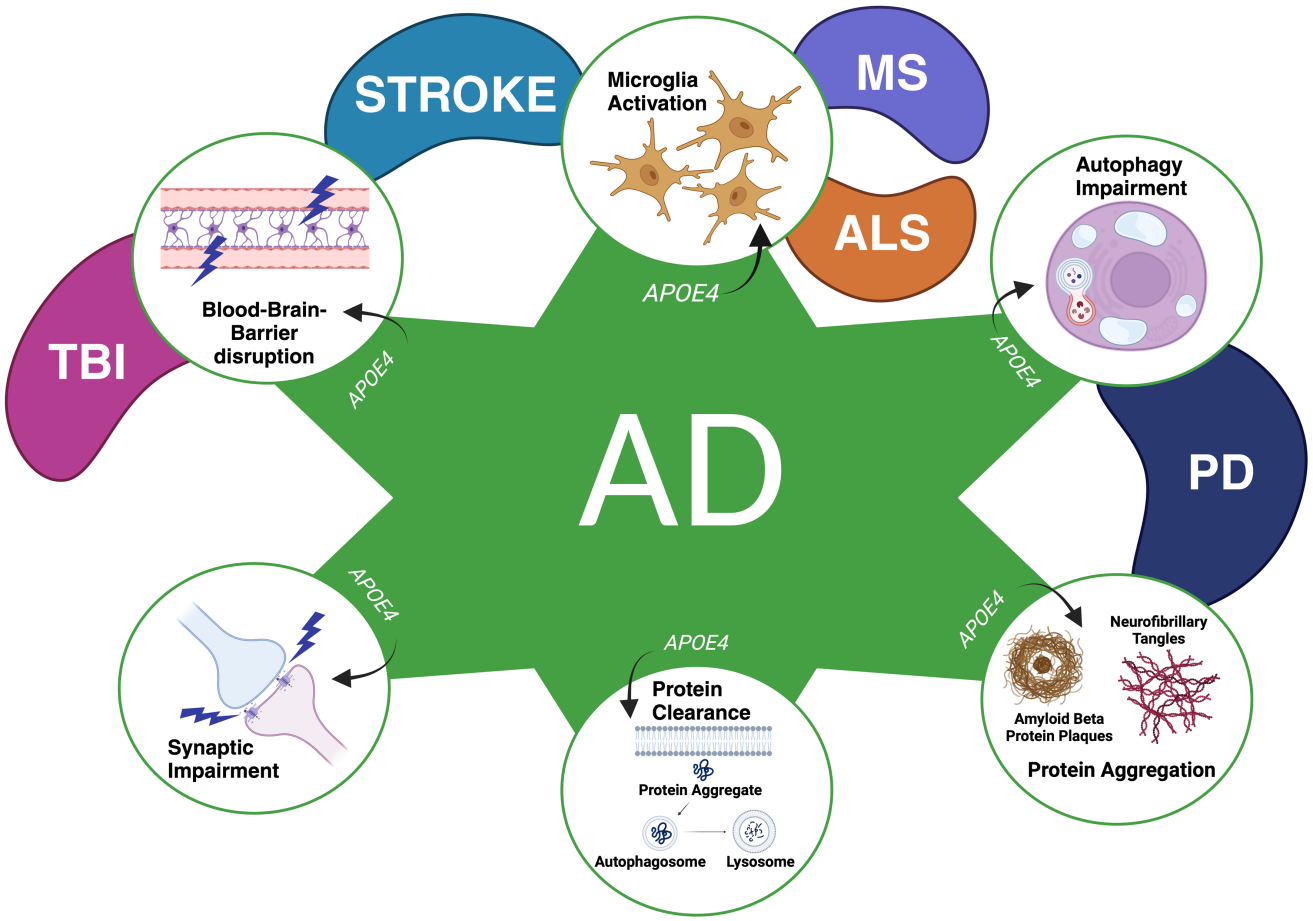
Mechanisms found to be influenced by the *APOE4* genotype in various CNS diseases thus far. A variety of mechanisms have been found to be influenced by *APOE4* in Alzheimer’s Disease (AD), Traumatic Brain Injury (TBI), Stroke, Multiple Sclerosis (MS), Amyotrophic Lateral Sclerosis (ALS), and Parkinson’s Disease (PD). *APOE4* has been found to contribute to enhanced synaptic impairment (AD), deficits in the clearance of proteins (AD), protein aggregations (PD and AD), autophagy impairment (AD and PD), disruptions in the blood–brain barrier (AD, TBI, Stroke) and the activation of microglia to promote neuroinflammation and alterations in microglial reactivity (AD, Stroke, MS, and ALS). Figure adapted from Fernández-Calle et al. 2022. Licensed under a Creative Commons Attribution 4.0 International License, http://creativecommons.org/licenses/by/4.0/. Figure was changed to focus on APOE4’s effect on specific neurodegenerative mechanisms as observed in previous literature.

Because of *APOE4’s* well-established prevalence in AD, its impact toward other CNS diseases has been investigated, including Amyotrophic Lateral Sclerosis (ALS), Multiple Sclerosis (MS), Traumatic Brain Injury (TBI), and PD (Fernández-Calle et al., 2022). Figure 1 illustrates mechanisms with *APOE4*-related neurodegeneration linked to the diseases, adapted from Fernández-Calle et al. (2022). Similarly in AD, *APOE4* may play a role in altering microglia activation to enhance neuroinflammation, disrupting the blood–brain barrier, influencing synaptic function, dysregulating protein clearance, increasing protein aggregations, and impairing autophagic processes in other neurodegenerative conditions as well (Krasemann et al., 2017; Main et al., 2018; Davis et al., 2020; Montagne et al., 2020; Fernández-Calle et al., 2022; Jin et al., 2022; Nechushtai et al., 2023). Given these shared mechanisms, further research onto how *APOE4* plays a role in these diseases may provide essential insight into its potential as both a clinical biomarker and a therapeutic target for them, especially for PD.

### 2.1 *APOE4* and Parkinsonism

Notably, clinically and pathologically, there is significant overlap between AD and PD, suggesting that they may have common underlying mechanisms. Both diseases contain similar pathological features, particularly protein aggregations: amyloid-beta plaques as well as neurofibrillary tangles in AD, as previously outlined, and Lewy bodies composed of alpha-synuclein protein in PD. Post-mortem analyses have revealed that these protein aggregations can co-occur in both diseases as well (Kalaitzakis et al., 2008; Burack et al., 2010; Irwin et al., 2012; Twohig and Nielsen, 2019; Visanji et al., 2019; Fernández-Calle et al., 2022). Approximately 50% of AD patients exhibit alpha-synuclein pathology, while up to 50% of PD patients with dementia have amyloid-beta proteins and neurofibrillary tangles that are sufficient enough for secondary AD diagnosis (Irwin et al., 2013; Lim et al., 2019). Moreover, both AD and PD also exhibit overlapping clinical symptoms, such as changes in cognitive function and extrapyramidal features (Morra and Donovick, 2014; Collins and Williams-Gray, 2016; Foguem and Manckoundia, 2018; Pierzchlińska et al., 2018; Wakasugi and Hanakawa, 2021). Considering these commonalities, the association between *APOE4* with PD and PD-related cognitive impairment has been debated over the years, with discussion that *APOE4* may potentially influence AD-type changes that contribute to the development of cognitive changes in PD subjects (Fernández-Calle et al., 2022; Haikal et al., 2024).

In the context of Parkinsonisms, the *APOE4* allele is prominently found in individuals with PD-MCI, PDD, and DLB, when compared to cognitively unimpaired healthy controls (HCs) and/or PD patients with normal cognition (PD-NC) (Parsian et al., 2002; Lashley et al., 2008; Tsuang et al., 2013; Outeiro et al., 2019). In PD, *APOE4* carriers are significantly more prevalent among those with PDD, with a 20–35% higher prevalence than in HCs and PD-NC subjects (Parsian et al., 2002; Williams-Gray et al., 2009; Tsuang et al., 2013). This association between PDD and *APOE4* is further supported by multiple studies demonstrating that those with PD and at least one *APOE4* allele have a greater likelihood in developing PDD when compared to those without the allele (Pankratz et al., 2006; Monsell et al., 2014; Huertas et al., 2017; Chung et al., 2021). Monsell et al. (2014) specifically reported a five-times higher risk for dementia within 5 years of diagnosis among PD *APOE4* carriers (Monsell et al., 2014). Interestingly, PD patients with *APOE4* also progress to PDD much earlier in comparison to non-carriers (Huertas et al., 2017; Tunold et al., 2021; Szwedo et al., 2022). Hence, *APOE4* may be a significant risk factor for dementia in PD and serve as a potential clinical biomarker for PDD. To note, individuals with PD who carry *APOE4* are diagnosed at a younger age compared to non-carriers as well, indicating that *APOE4* may also be a significant predictor for early onset PD (Pankratz et al., 2006; Szwedo et al., 2022).

In terms of PD-related cognitive impairment, PD patients who are *APOE4* carriers show greater cognitive impairment (Pavlova et al., 2014; Paul et al., 2016; Chung et al., 2021). Tropea et al. (2018) report a 3.6 fold increased risk of cognitive impairment in PD *APOE4* carriers when compared to non-carriers (Tropea et al., 2018). Additionally, Szwedo et al. (2022) observed that PD *APOE4* carriers demonstrate an accelerated rate of cognitive impairment over a 10-year-follow up (Szwedo et al., 2022). Thus, this indicates that *APOE4* may play an essential role in facilitating an enhanced likelihood and faster progression of cognitive impairment in those with PD. Further, PD patients with *APOE4* also exhibit a decline in specific cognitive processes. This includes executive and visuospatial function, impaired short-term memory, language, and attention, contrasting to patients without the polymorphism and/or HCs (Mata et al., 2014; Pavlova et al., 2014; Paul et al., 2016; Samat et al., 2017; Myers et al., 2022). Studies utilizing the Mattis Dementia Rating Scale-2 (DRS) for neuropsychological evaluation also demonstrate that PD *APOE4* carriers have a greater decline in distinct cognitive abilities, including conceptualization, construction, initiation, and overall memory performance when compared to those without the allele (Morley et al., 2012; Tropea et al., 2018). Therefore, this may also suggest that *APOE4* adversely impacts certain cognitive processes in individuals with PD.

Despite this evidence, some studies have failed to find a link between *APOE4* and cognitive impairment in PD (Ezquerra et al., 2008; Kurz et al., 2009; Williams-Gray et al., 2009; Federoff et al., 2012). For instance, Federoff et al. (2012) found no relationship between memory loss at PD diagnosis and *APOE4* amongst a large PD cohort (Federoff et al., 2012). Similarly, an observational cohort study by Mengel et al. (2016) found that *APOE4* was not associated with the diagnosis of PD-MCI and PDD, nor with declines in specific neuropsychological domains in PD subjects (Mengel et al., 2016). Several studies also reported no association between *APOE4* carrier status and PDD (Ezquerra et al., 2008; Kurz et al., 2009; Williams-Gray et al., 2009). Some findings have shown that possessing the polymorphism does not affect the risk, development, and progression to dementia, as well as the overall rate of cognitive impairment in PD instead (Ezquerra et al., 2008; Kurz et al., 2009; Williams-Gray et al., 2009). Discrepancies in the literature have been attributed to several factors. For example, small sample sizes resulting in low statistical power have been noted to weaken findings on the association between *APOE4* and cognitive impairment in PD (Kurz et al., 2009; Federoff et al., 2012). In addition, results from cross-sectional studies have been discussed to potentially be misleading, as the effect of *APOE4* may be time-dependent in PD by emerging later in disease progression like in AD (Collins and Williams-Gray, 2016). Studies that did not find an association between *APOE4* and cognitive impairment in their cohorts have emphasized the need for longitudinal studies to further evaluate the effect of *APOE4* and cognitive function in PD, which are currently limited (Ezquerra et al., 2008; Collins and Williams-Gray, 2016; Mengel et al., 2016; Zhu et al., 2024). It should be noted however that these studies are outdated, and over the past 5 years, an increasing number of research demonstrate that *APOE4* in PD patients is indeed linked to a greater risk in cognitive impairment, a decline in specific cognitive processes, and serves as a predictor for PDD, with an earlier and more rapid onset (Rawle et al., 2018; D’Souza and Rajkumar, 2020; Tan et al., 2021; Tipton et al., 2021; Tunold et al., 2021; Myers et al., 2022; Okubadejo et al., 2022; Szwedo et al., 2022; Umeh et al., 2022; Chen et al., 2023; Liu et al., 2023; Real et al., 2023; Zenuni et al., 2023). Thus, understanding the role of *APOE4* toward PD-related cognitive impairment may be worthwhile due to growing evidence showing its association with such non-motor symptom.

In the context of DLB, it is also rationalized that *APOE4* may play a role in its pathogenesis, and a strong risk factor of the disease (Tsuang et al., 2013; Rongve et al., 2019; Kaivola et al., 2021). Genome-wide association studies (GWAS) examined *APOE4* to be a significant loci linked to DLB (Bras et al., 2014; Guerreiro et al., 2018; Rongve et al., 2019; Chia et al., 2021; Kaivola et al., 2021; Bousiges et al., 2023). A GWAS study of 828 clinically diagnosed DLB subjects showed that *APOE4* alongside the *GBA* gene was significantly associated with higher DLB risk (Rongve et al., 2019). Another GWAS study including 495 DLB cases showed that *APOE4* was significantly associated in DLB subjects with AD-co-pathology when compared to those with no-co-pathology, potentially highlighting the relevance of *APOE4* and its relationship to AD-like changes in DLB (Kaivola et al., 2021). Similar to PDD and AD, neuropathological studies show that DLB subjects also exhibit amyloid-beta and tau pathology co-occurring with alpha-synuclein pathology, where approximately 40–70% harbor amyloid-beta plaques across studies (Vrillon et al., 2024). DLB subjects also show decreases in Cerebrospinal (CSF) biofluid markers that measure amyloid-beta and tau proteins, indicating that amyloid and tau metabolism may be disrupted in DLB as well (van Steenoven et al., 2019; Bolsewig et al., 2024; Vrillon et al., 2024). Thus, this may suggest that *APOE4* is also critical in DLB pathogenesis, especially in contributing to AD-like pathology to exacerbate cognitive impairment. Furthermore, more pronounced cognitive impairment is observed in DLB *APOE4* carriers when compared to non-carriers, including in attention, learning, memory, and executive function (Ballard et al., 2001; Mirza et al., 2019; Nasri et al., 2022). Therefore, further reinforcing *APOE4*’s potential role in cognitive impairment in those with DLB.

Overall, in the current literature, mechanisms involving *APOE4* that may be associated with cognitive impairment in PD and DLB is less understood. However, a possible mechanism may include enhancing the accumulation of alpha-synuclein aggregations in Lewy bodies (McKeith et al., 2005; Davis et al., 2020; Zhao et al., 2020; Attems et al., 2021; Haikal et al., 2024). This is supported with preclinical studies using alpha-synuclein mice models, which examined that those expressing the allele developed the most burden of alpha-synuclein pathology, as well as greater and faster cognitive impairment, including memory dysfunction, when compared to those with *APOE2* and *APOE3* (Davis et al., 2020; Zhao et al., 2020). It has been proposed that this relation between *APOE4* and enhanced alpha-synuclein pathology may be due to various factors. APOE4’s role in the disruption of cholesterol homeostasis has been discussed, considering that one of the preclinical studies by Zhao et al. (2020) observed in their APOE4 cerebral organoids to have both increases in alpha-synuclein and lipid droplet accumulation (Zhao et al., 2020). Another alternative explanation may involve alpha-synuclein directly interacting and co-aggregating with APOE, as observed in post-mortem studies, suggested to be accelerated at the presence of APOE4 (Rohn and Mack, 2018; Paslawski et al., 2019; Davis et al., 2020; Zhao et al., 2020). However, further research is needed to characterize the cellular and molecular processes involving *APOE4* that may exacerbate alpha-synuclein pathology (Davis et al., 2020). Other mechanisms may include *APOE4*’s role in enhancing amyloid-beta pathology, as established in AD, due to the significant prevalence in amyloid-beta in the brains of both DLB and PDD subjects. However, a study by Tsuang et al. (2013) examined a high proportion of individuals with *APOE4* among those with PDD and pure DLB, a type of DLB characterized by the presence of Lewy bodies without AD-like pathology, but they exhibited low amyloid-beta plaques. This is consistent with other studies analyzing no association between *APOE4* and pure DLB (Dickson et al., 2018; Kaivola et al., 2021). Therefore, *APOE4* may also play a role in amyloid-independent mechanisms in PD and DLB to induce neurodegeneration and cognitive changes, just like in AD.

## 3 Imaging *APOE4’s* effect in Parkinsonism

Neuroimaging techniques aid the understanding of how changes in the brain may be associated with mental, behavioral, and cognitive processes (Yen et al., 2023). Herein, we will review neuroimaging studies conducted in humans to determine the effect of *APOE4* on brain changes that may influence cognitive function in people with PD, PD-MCI, and PDD. Considering that *APOE4* is associated with DLB risk, and that DLB *APOE4* carriers also exhibit greater cognitive impairment than non-carriers, we also review studies that investigate the effect of *APOE4* toward the brains of DLB subjects (Ballard et al., 2001; Boot et al., 2013; Tsuang et al., 2013; Guerreiro et al., 2018; Mirza et al., 2019; Rongve et al., 2019; Kaivola et al., 2021; Nasri et al., 2022). Imaging metrics, cognitive function, and genetic status will be evaluated and discussed to determine potential relationships.

### 3.1 *APOE4* and amyloid-beta protein pathology

Abnormal accumulations of amyloid-beta proteins have been observed in the brains of subjects with PD-related cognitive impairment (Gomperts et al., 2013; O’Callaghan and Lewis, 2017). Several studies have reported higher levels of amyloid-beta proteins in individuals with PD-MCI and PDD when compared to HCs and PD-NC subjects (Irwin et al., 2012; Gomperts et al., 2013; Hepp et al., 2016). These increases in amyloid-beta in PD subjects with cognitive impairment is also observed in specific brain areas, including the temporal, parietal, and frontal regions (Maetzler et al., 2008; Hepp et al., 2016; Mihaescu et al., 2022).

Pre-clinical and clinical research have shown that *APOE4* carriers have higher amyloid-beta protein accumulation, as observed in AD subjects and transgenic mice (Liu et al., 2013; Kanekiyo et al., 2014). Similarly, this association between *APOE4* and enhanced amyloid-beta accumulation is also observed in PD. Various Positron Emission Tomography (PET) studies using radiotracers that measure these proteins in the brain, including [11C]-Pittsburgh Compound B (PiB), [18F]-Florbetaben (FBB), or [18F]-florbetapir ([18F]-AV-45), revealed significant increased retention and standardized uptake value ratios (SUVR) in PD *APOE4* carriers than non-carriers (See Table 1 for more details) (Maetzler et al., 2009; Gomperts et al., 2012; Vijayaraghavan et al., 2014; Akhtar et al., 2017; Jung et al., 2021). Notably, these studies showed that higher retention and SUVR values for amyloid-beta proteins in specific brain regions was associated with *APOE4* in PD subjects with mild cognitive impairment and/or dementia, highlighting *APOE4’*s potential role in influencing enhanced regional amyloid-beta pathology. For instance, Gomperts et al. (2012) discovered that the number of *APOE4* alleles (0, 1, 2) was correlated with amyloid-beta protein within the precuneus (via [11C]-PiB PET) across their PD-MCI, PDD, DLB, and HC groups (Gomperts et al., 2012). This brain region has been associated with memory deficits when containing higher amyloid-beta protein depositions (Perrotin et al., 2012; Farrell et al., 2018; Fang et al., 2020). However, the authors did not find a significant correlation between precuneus amyloid-beta and cognitive function, as measured by the Mini-Mental State Examination (MMSE), in their PD-MCI (mean age = 69.4) and PDD groups (mean age = 71.7) (Gomperts et al., 2012). It was discussed that this may be due to the possibility that other pathological mechanisms could underlie the progression of PDD, rather than amyloid-beta burden (Gomperts et al., 2012). Such rationale may align with findings that amyloid-beta deposition is not well-correlated with cognitive impairment in older adults, and that other abnormalities known to be more strongly correlated with cognitive changes may be the reason for cognitive impairment instead, such as tau pathologies (Villemagne et al., 2013; Jack et al., 2014; Jansen et al., 2015; Bejanin et al., 2017; Brookmeyer and Abdalla, 2018; Ramanan et al., 2021). Low-levels of amyloid-beta burden in their PDD group was also discussed to potentially affect their ability to robustly detect an association between amyloid levels and cognitive function (Gomperts et al., 2012). Correspondingly, Maetzler et al. (2009) identified that all their subjects positive for amyloid-beta (via [11C]-PiB PET), measured in their frontal brain regions, posterior cingulate, as well as in superior parietal, lateral temporal and occipital lobe, had dementia (PDD and DLB) and a higher prevalence of *APOE4* when compared to non-demented and demented subjects negative for [11C]-PiB (Maetzler et al., 2009). This may support *APOE4*’s association with dementia in PD and DLB, as previously discussed, which may be explained by enhanced amyloid-beta pathology in specific brain regions. Similarly, Akhtar et al. (2017) also found that higher amyloid-beta (via [18F]-AV-45 PET SUVR) in memory-related brain areas, including the anterior and posterior cingulate, precuneus, as well as temporal, frontal and parietal cortices, was associated with *APOE4* in their PD-MCI and PD-NC groups when controlling for age (Akhtar et al., 2017). This may indicate that *APOE4* enhances amyloid-beta burden in regions essential for cognitive domains affected in those with PD. Moreover, Vijayaraghavan et al. (2014) observed in their combined sample of *APOE4* carriers (PD-NC, PD-MCI, PDD, and DLB) to have higher amyloid-beta (assessed via [11C]-PiB retention) in their striatal and cortical regions, as well as a lower MMSE score when compared to non-carriers (Vijayaraghavan et al., 2014). Jung et al. (2021) also found using PET with [18F]-FBB that typical LBD was linked to increased occipital beta-amyloid burden though its interaction with *APOE4*, as analyzed in their combined group including subjects with DLB with amyloid and pure LBD (PD and DLB). However, it should be noted that the heterogeneity of the samples in these two studies since they combined subjects with distinct Parkinsonisms and different stages of cognitive impairment may complicate interpretations. Combining PD subjects with varying cognitive levels may make it challenging to interpret the relationships between *APOE4*, cognition, and amyloid-beta accumulation in PD, since a previous systematic review of 11 PiB studies analyzed that each cognitive stage differs in amyloid-beta burden (Petrou et al., 2015). Including DLB subjects alongside PD subjects may further introduce complexity, given the distinct cognitive profiles and trajectories of DLB and PD (Aarsland et al., 2021). Overall, all these studies suggests that *APOE4* may influence cognitive impairment in those with PD by contributing to region-specific amyloid-beta protein burden (Maetzler et al., 2008; Hepp et al., 2016; Mihaescu et al., 2022). Though the brain regions differ across studies, it is apparent that higher retention and SUVR values are found in areas known to play a role in cognitive processes impaired in PD subjects.

**Table 1:** Details of studies evaluating amyloid-beta protein burden including parkinsonian subjects (PD and DLB) with *APOE4* genotype.

Reference	Neuroimaging method	Clinical population	N of *APOE4+*	N of APOE4−	Sex (M/F)	Summary
Vijayaraghavan et al. (2014)	[11C]-PiB PET	9 DLB	6	3	7/2	Significantly greater [11C]-PiB retention levels in cortical and striatal regions, and lower MMSE scores in *APOE4* carriers than non-carriers Retention levels measured in dorsolateral, prefrontal, ventrolateral prefrontal, and orbitofrontal regions; anterior cingulate; superior parietal, lateral temporal, and lateral occipital lobes; and posterior cingulate
		10 PDD	3	7	4/6	
		10 PDND	1	8	8/2	
		5 HC	–	5	2/3	
Maetzler et al. (2009)	[11C]-PiB PET	9 DLB	6	2	33.3% F	all PiB+ subjects were demented (DLB and PDD), had higher prevalence of *APOE4*, and lower MMSE scores when compared to PiB- subjects PiB+ subjects had increased [11C]-PiB uptake in posterior cingulate, cuneus, precuneus, striatum temporoparietooccipital cortex, and frontal cortex
		12 PDD	4	7	50% F	
		14 PDND	2	10	20.4% F	
Jung et al. (2021)	[18F]-FBB PET	126 NC	17.5% *APOE4* carriers; 0 *APOE4+/+*	22 *APOE2* carriers	91 F	Interaction between *APOE4* and typical LBD (studied in pLBD and DLB with amyloid subjects) was associated with worser Clinical Dementia Rating Sum of Boxes scores and increased occipital [18F]-FBB SUVR
		21 DLB with amyloid	47.6% *APOE4* carriers, including DLB with amyloid; 0 *APOE4+/+*	2 *APOE2* carriers	8 F	
		56 pLBD (PD and DLB)	19.6% *APOE4* carriers; 1 *APOE4+/+*	9 *APOE2* carriers	33 F	
Gomperts et al. (2012)	[11C]-PiB PET	18 DLB	8	5	17/1	Number of *APOE4* alleles positively correlated with amount of [11C]-PiB burden in precuneus of HCs, PD-MCI, PDD, and DLB groups Having at least one *APOE4* allele was associated with higher precuneus [11C]-PiB retention by 2-fold when adjusting for diagnostic group and age Precuneus [11C]-PiB uptake was not significantly related to MMSE scores for any of the PD-NC, PD-MCI, PDD, or HCs groups
		12 PDD	5	6	10/2	
		14 PD-MCI	2	11	11/3	
		29 PD	6	22	20/9	
		85 HC	12	52	30/55	
Akhtar et al. (2017)	[18F]-AV-45-PET	19 PD-MCI	36.8%	–	73.8% M	17 subjects positive for [18F]-AV-45-PET Subjects with *APOE4* had higher averaged [18F]-AV-45 SUVR values compared to non-carriers when controlling for age Average SUVRs measured in anterior cingulate, posterior cingulate, precuneus, temporal cortex, frontal cortex, and parietal cortex
		42 PD-NC	31.0%	–	68.4% M	
Gomperts et al. (2013)	[11C]-PiB PET	46 PD	–	–	33/13	*APOE4* allele not strongly associated with [11C]-PiB reuptake [11C]-PiB reuptake values calculated in precuneus, frontal cortex, and striatum
		35 PD-NC	–	–	24/11	
		11 PD-MCI	–	–	9/2	
Mashima et al. (2017)	[18F]-FBB PET	33 PD	2 *APOE3/4,* 1 *APOE4+/+*	1 *APOE2/3*	22/11	*APOE4* carriers and noncarriers negative for amyloid-beta protein deposition when analyzing [18F]-FBB binding in lateral temporal, frontal and parietal lobes, as well as posterior cingulate cortex and precuneus
Campbell et al. (2013)	[11C]-PiB PET	53 PD	13	28	44/9	Measurements of mean [11C]-PiB binding using Principal Component Analysis in PD participants did not associate with *APOE4* status
		27 HC	2	20	5/22	
Rowe et al. (2007)	[11C]-PiB PET	27 HC	1 *APOE2/4,* 6 *APOE3/4,* 1 *APOE 4+/+*	6 *APOE2/3*, 13 *APOE3/3*	14/13	Pooled cohort including HC and DLB subjects showed significant association between *APOE4* and higher [11C]-PiB binding No significant association between [11C]-PiB binding and *APOE4* status when comparing *APOE4* carriers and non-carriers within DLB group
		10 DLB	6 *APOE3/4,* 1 *APOE4+/+*	2 *APOE3/3*	8/2	
Nedelska et al. (2019)	[11C]-PiB PET	140 CU	64	–	88.6% M	Probable DLB *APOE4* carriers showed higher baseline [11C]-PiB SUVR than non-carriers No differences in the change of [11C]-PiB SUVR in probable DLB *APOE4* carriers vs. non-carriers from baseline to follow-up [11C]-PiB SUVR calculated in parietal, posterior cingulate, precuneus, prefrontal, orbitofrontal, temporal, and anterior cingulate cortices
		35 probable DLB	16	–	88.6% M	
Ferreira et al. (2020)	[11C]-PiB PET	417 probable DLB; 367 from European DLB consortium data, 50 from Mayo Clinic Cohort	122	135	287/129	*APOE4* carriers more commonly found in subjects positive for amyloid-beta protein biomarkers *APOE4* carriers had more enhanced amyloid-beta protein burden at younger ages than non-carriers -[11C]-PiB retention and SUVR measured in bilateral parietal, temporal, prefrontal, orbitofrontal, and anterior cingulate areas

APOE4, Apolipoprotein E4; APOE2, Apolipoprotein E2; APOE3, Apolipoprotein E3; APOE4+/+, APOE4 homozygous carriers (have two APOE4 alleles); N of APOE4 +, Number of individuals who have at least one APOE4 allele; N of APOE4 −, Number of individuals who do not have at least one APOE4 allele; DLB, Dementia with Lewy Bodies; CU, Cognitively Unimpaired Controls; HC, Healthy Controls; PD, Parkinson’s Disease; PDND, PD with no Dementia; PDD, PD with Dementia; PD-NC, PD with normal cognition; PD-MCI, PD with Mild Cognitive Impairment; DLB-MCI, Dementia with Lewy Bodies and Mild Cognitive Impairment; NC, Normal Cognition Controls; LBD, Lewy Body Disease; pLBD, Pure Lewy Body Disease (PD + DLB); PET, Positron Emission Tomography; [11C]-PiB, [11C]-Pittsburgh Compound B; [11C]-PiB-PET, [11C]-Pittsburgh compound B-Positron Emission Tomography; PiB+, Positive for [11C]-Pittsburgh Compound B in the brain; PiB−, Negative for [11C]-Pittsburgh Compound B in the brain; [18F]-FBB, [18F]-Florbetaben; [18F]-FBB PET, [18F]-Florbetaben Positron Emission Tomography; [18F]-AV-45, [18F]-Florbetapir; [18F]-AV-45-PET, [18F]-Florbetapir Positron Emission Tomography; SUVR, Standardized Uptake Value Ratio; MMSE, Mini-Mental Standardized Examination; M, males; F, females.

In contrast, several studies have reported no association between *APOE4* and amyloid-beta protein burden instead. A study by Campbell et al. (2013) using principal component analysis provided evidence that *APOE4* status did not affect amyloid-beta burden (assessed via mean [11C]-PiB binding) in 13 PD subjects experiencing cognitive impairment (Campbell et al., 2013). Mashima et al. (2017) also found that PD *APOE4* carriers and non-carriers were both negative for amyloid-beta protein pathology using PET with [18F]-FBB (Mashima et al., 2017). However, the analysis of only 3 carriers and 1 non-carrier in a total of 33 subjects may lack statistical power, in contrast to larger studies showing a higher prevalence of amyloid-beta protein pathology in PD individuals with *APOE4* as previously discussed (Maetzler et al., 2008; Gomperts et al., 2012; Vijayaraghavan et al., 2014; Akhtar et al., 2017; Mashima et al., 2017). Similarly, Gomperts et al. (2013) study claimed that *APOE4* was not strongly associated with amyloid-beta burden (via [11C]-PiB) in their PD group, including those with PD-MCI, but proposed that such observations may be due to their sample sizes (*n* = 46). Observed discrepancies amongst PiB studies may be attributed to the types of groups analyzed and their levels of cognitive severity. Some studies identifying a significant association between *APOE4* and amyloid-beta burden included cohorts with more severe cognitive impairment (i.e., classified to have DLB and PDD), whereas all the studies finding no association included participants with PD-MCI or PD without dementia only (Maetzler et al., 2009; Gomperts et al., 2012, 2013; Campbell et al., 2013; Vijayaraghavan et al., 2014; Mashima et al., 2017). This indicates that the severity of cognitive impairment may potentially play a critical role in the relationship between *APOE4* status and amyloid-beta accumulation in PD. Discrepancies may also be attributed to the presence of co-morbidities of AD and PD protein pathology, as well as their co-localization with neuroinflammation, which may complicate the accurate measurement of amyloid-beta proteins. Individuals with advanced PD are more likely to exhibit overlapping protein pathologies, as both AD and PD pathologies commonly occur at later stages of the disease (Visanji et al., 2019). Similarly, enhanced neuroinflammation, measured through reactive astrocytes, has been shown to increase with age in DLB and PDD subjects, highlighting the prevalence of neuroinflammation in later stages of the disease also (van den Berge et al., 2012). Thus, since some significant studies included individuals with advance stages of the disease (PDD and DLB) while studies finding no significant association included none, it is also possible that prevalence of heightened co-pathology and neuroinflammation may potentially influence radiotracer uptake. This is further supported with evidence showing that PiB can bind to Lewy bodies and neurofibrillary tangles *in vivo* (Fodero-Tavoletti et al., 2007; Lockhart et al., 2007).

Furthermore, higher amyloid-beta protein pathology is potentially associated with greater impaired cognition in DLB subjects (Nelson et al., 2009; Gomperts et al., 2012). However, several studies reported that DLB *APOE4* carriers had higher amyloid burden (via [11C]-PiB) than non-carriers, while others did not (See Table 1) (Rowe et al., 2007; Maetzler et al., 2009; Gomperts et al., 2012; Vijayaraghavan et al., 2014; Nedelska et al., 2019; Ferreira et al., 2020; Jung et al., 2021). Thus, further research is warranted to mitigate these inconsistencies to make robust conclusions on how *APOE4* influences DLB-related cognitive impairment via increasing amyloid-beta proteins also.

### 3.2 *APOE4* and structural brain changes in Parkinsonian subjects

Numerous studies, summarized in Table 2, have investigated the relationship between *APOE4* and structural brain measures, including those that quantify gray and white matter, as well as ventricular size, in those with PD and DLB.

**Table 2:** Details of studies evaluating structural brain changes including parkinsonian subjects (PD and DLB) with *APOE4* genotype.

Reference	Neuroimaging method	Structural brain changes analyzed	Clinical population	N of *APOE4+*	N of APOE4−	Sex (M/F)	Summary
Kandel et al. (2016)	T_1_-weighted MRI and FLAIR MRI	WMH	58 cognitively normal PD subjects	12	–	35 M	*APOE4* carriers in PPMI cohort had greater WMH volumes when compared to *APOE4* carriers in the ADNI cohort
Mirza et al. (2019)	T_1_-weighted, T_2_-weighted, and Proton Density-weighted MRI	WMH	50 DLB	20 *APOE4*+/−, 8 *APOE4*+/+	22	–	Pooled sample of *APOE4* carriers including DLB subjects had greater WMH volumes that was associated with worsened attention, executive function, learning, memory, and language when compared to non-carriers
Ferreira et al. (2023)	T_1_-weighted and T_2_-weighted FLAIR MRI	WMH	30 DLB	12	–	26 M	Absence of *APOE4* predicted greater WMH volumes Higher WMH volumes predicted lower gray matter volume in the frontal orbital, frontal medial, and inferior temporal cortices
			100 CU	30	–	85 M	
Barber et al. (1999)	T_1_-weighted, T_2_-weighted, and Proton Density-weighted MRI	WMH	22 DLB	0.30 allele frequency of *APOE4*	0.68 *APOE3* allele frequency, 0.02 *APOE2* allele frequency	14/8	No significant association between *APOE4* and WMH when comparing subjects with and without *APOE4*, including late-onset DLB group
Rane et al. (2018)	Structural MRI Data	Cortical Thickness	33 PD-MCI	4	29	22 M	Significantly reduced cortical thickness in middle temporal gyrus, inferior parietal region, and precuneus of *APOE4* carriers with aMCI when compared to non-carriers within the group
			28 cognitively normal controls	8	20	18 M	
Sampedro et al. (2019)	T_1_-weighted MRI	Cortical Thickness	93 PD-NC	30%	–	37% F	*APOE4* carriers with BDNF Val/Met genotype reported to have significant cortical thinning in posterior cortical regions for both HC and PD-NC
			38 HC	33%	–	32% F	
Nicoletti et al. (2016)	T_1_-weighted MRI, Voxel-Based Morphometry analysis	Brain Volume	49 PD	25 (13 PDD)	24 (12 PDD)	11/14 *APOE4*+, 10/14 *APOE4*−	No significant association of *APOE4* and reduced gray matter volume in PD patients, either with or without dementia No differences in shape of deep gray matter structures between PD patients with or without *APOE4*, including the hippocampus, thalamus, and caudate, and globus pallidus
			26 HC	–	–	11/15	
Barber et al. (2000)	T_1_-weighted Volumetric MRI	Brain Volume	27 DLB	–	–	19/8	No significant differences in volumetric indices between subjects with and without *APOE4*, including those in the DLB group Volumetric indices measured for whole-brain, ventricles, frontal lobe, temporal lobe, hippocampus, and amygdala
			26 HC	–	–	14/12	
Saeed et al. (2018)	T_1_-weighted Structural MRI	Brain Volume	48 DLB	20 *APOE4*+/−, 7 *APOE4*+/+	21	32 M	*APOE4* is inversely associated with hippocampal volume in a dose-dependent manner both in a pooled sample that includes a DLB group and in an analysis stratifying the DLB group; (1) *APOE4*+/+ = smaller hippocampal volume, (2) *APOE4*+/− = intermediate hippocampal volume, (3) *APOE4*−/− = larger hippocampal volume Reduced hippocampal volume associated with reduced scores in California Verbal Learning Total Recall Test
Apostolova et al. (2012)	T_1_-weighted MRI	Ventricular Enlargement and Cortical Thickness	127 PD-CN	48	–	74/53	*APOE4* not a predictor for smaller right hippocampal radial distance in PD-MCI group *APOE4* not a predictor for smaller right hippocampal radial distance in PD-CN when comparing to NC Only trend-level association between *APOE4* and larger bilateral temporal, occipital, and left frontal horn radial distance of the lateral ventricles in the PD-MCI group relative to PD-CN
			31 PD-MCI	7	–	19/12	
			100 NC	32	–	48/52	
Beyer et al. (2013)	T_1_-weighted MRI	Ventricular Enlargement	18 PD-MCI	4	–	10/8	*APOE4* status did not show a significant association with ventricular radial distance in pooled sample of PD-MCI and PD-CN subjects
			73 PD-CN	28	–	50/23	

APOE4, Apolipoprotein E4; APOE2, Apolipoprotein E2; APOE3, Apolipoprotein E3; APOE4+/+, APOE4 homozygous carriers (two APOE4 alleles); APOE4 +/−, APOE4 heterozygous carriers (one APOE4 allele); APOE4−/−, APOE4 non-carriers (No APOE4 allele); N of APOE4 +, Number of individuals who have at least one APOE4 allele; N of APOE4 −, Number of individuals who do not have at least one APOE4 allele; DLB, Dementia with Lewy Bodies; CU, Cognitively Unimpaired Controls; HC, Healthy Controls; NC, Normal Cognition Controls; PD, Parkinson’s Disease; PDD, PD with Dementia; PD-NC/PD-CN, PD subjects with normal cognition/who are cognitively normal; PD-MCI, PD with Mild Cognitive Impairment; aMCI, amnestic Mild Cognitive Impairment; WMH, White Matter Hyperintensities; MRI, Magnetic Resonance Imaging; FLAIR, Fluid-Attenuated Inversion Recovery Sequence; BDNF val/met, Brain-derived neurotrophic factor valine to methionine polymorphism; PPMI, Parkinson’s Progression Markers Initiative; ADNI, Alzheimer’s Disease Neuroimaging Initiative; M, males; F, females.

Measurements known to help assess gray matter changes have been explored to be associated with PD-related cognitive impairment. This includes cortical thickness, where its reduction has been examined in PD-MCI and PDD subjects using Magnetic Resonance Imaging (MRI) (Segura et al., 2014; Koshimori et al., 2015; O’Callaghan and Lewis, 2017; Gasca-Salas et al., 2019), and is proposed to be a potential marker for the conversion from PD-MCI to PDD (Segura et al., 2014; Chung et al., 2019). Two studies identified a relationship between *APOE4* and decreases in cortical thickness of specific regions of PD patients, especially within posterior cortical areas, including in the temporal and parietal regions (Rane et al., 2018; Sampedro et al., 2019). Rane et al. (2018) showed that 28 *APOE4* carriers (24 AD and 4 PD) who had amnestic MCI (aMCI) showed significant decreases in the cortical thickness of the middle temporal gyrus when compared to non-carriers, a brain region essential for language, visual perception, and episodic memory processing (Onitsuka et al., 2004; Rane et al., 2018). This is consistent with previous literature showing that the middle temporal gyrus is atrophied and thinner in those with PD-MCI when compared to PD subjects without cognitive impairments or HCs (Zhang et al., 2015; Hanganu and Monchi, 2016). Rane et al. (2018) also examined cortical thinning in the parietal and precuneus regions in their *APOE4* carriers, areas previously found to be affected in PD individuals experiencing cognitive impairment and are also essential for memory function (Perrotin et al., 2012; O’Callaghan and Lewis, 2017; Farrell et al., 2018; Rane et al., 2018; Jawabri and Sharma, 2023). The results of Rane et al.’s (2018) study may be challenging to generalize however, as only a small sample size of PD subjects within their *APOE4* group was observed (Rane et al., 2018). Furthermore, Sampedro et al. (2019) reported that an interaction between *APOE4* and a specific genotype for the *Brain Derived Neurotrophic Factor (BDNF)* was associated with greater cortical thinning in posterior cortical regions in both their PD-NC and HC groups (Sampedro et al., 2019). These results may also suggest that a combination of genetic factors, including *APOE4*, may contribute to reduced cortical thickness of specific regions in PD that may potentially predict cognitive impairment. This is supported by evidence examining that posterior-cortical degeneration in PD is associated with more severe cognitive impairment in PD and is strongly associated with PDD (Pagonabarraga and Kulisevsky, 2012; Uribe et al., 2018). Collectively, we infer that both studies indicate that *APOE4* influences reduced cortical thickness in regions essential for cognitive function, potentially contributing to future cognitive changes in those with PD. However, findings from Apostolova et al. (2012) may contradict this, as the thickness of the hippocampus, a region well-known for memory function, was not significantly affected by *APOE4* status in PD patients instead, despite strong positive correlations with MMSE score and cognitive status (PD vs. PD-MCI) (Apostolova et al., 2012). Yet, methodological differences in thickness analysis, such as the use of radial distance measured by Apostolova et al. (2012) versus the FreeSurfer pipeline in the two studies, may have impacted sensitivity in examining *APOE4*-related thickness changes to contribute to these discrepancies (Apostolova et al., 2012; Rane et al., 2018; Sampedro et al., 2019).

Decreases in gray matter volume (GMV) have also been a widely examined gray matter change to be associated with PD-related cognitive impairment (Xia et al., 2013; Noh et al., 2014; O’Callaghan and Lewis, 2017; Hou and Shang, 2022). Reduced GMV was detected in PD patients with cognitive deficits, and associated with worsened performance in the Montreal Cognitive Assessment (MoCA) and MMSE (Xia et al., 2013; Noh et al., 2014; O’Callaghan and Lewis, 2017; Li et al., 2022). A meta-analysis by Xu et al. (2016) also found that individuals with PD-MCI and PDD had greater decreases in GMV when compared to PD patients without cognitive impairments, including in the hippocampus, insula, left superior and inferior temporal lobe, and left superior frontal lobe (Xu et al., 2016). There is limited data however with regards to the association of *APOE4* and GMV alterations in PD. One study by Nicoletti et al. (2016) reported no significant differences in whole brain GMV when comparing PD patients with and without *APOE4*, regardless of dementia status (Nicoletti et al., 2016). They also found no significant differences in the shape of deep gray matter structures between PD *APOE4* carriers and non-carriers, including the hippocampus, thalamus, globus pallidus, and caudate nucleus (Nicoletti et al., 2016). This may indicate that *APOE4* may not influence GMV changes relevant in PD individuals with cognitive impairment. However, caution is necessary as these conclusions are derived from a single study. Since there are existing studies that do observe a relationship between *APOE4* and GMV alterations in regions relevant for cognitive function, it may be worthwhile to conduct more research in PD to further evaluate *APOE4*’s association with GMV (Spampinato et al., 2011; Haller et al., 2017; Cacciaglia et al., 2018). In terms of DLB, there is conflicting evidence with regards to the association of *APOE4* and hippocampal GMV, a brain region moderately atrophied in those with the disease (Chow et al., 2012). Saeed et al. (2018) studied in their combined group of *APOE4* carriers with DLB and AD, as well as after stratifying the two groups that *APOE4* was linked to hippocampal volume loss, with two alleles causing greater atrophy than one or none, and that this reduction in volume was associated with impaired learning performance (Saeed et al., 2018). However, Barber et al. (2000) found no differences in volumetric measurements, including the hippocampus, of DLB patients when comparing those with and without *APOE4* (Barber et al., 2000). Variations in analyzing volume measurements may influence the observed differences between studies, particularly given that each employed different imaging software (Barber et al., 2000; Saeed et al., 2018). Saeed et al. (2018) also offered a thorough characterization of their sample, detailing the characteristics of both *APOE4* carriers and non-carriers, which may further strengthen their findings (Saeed et al., 2018). Contrastingly, Barber et al. (2000) lacked demographic information for their *APOE4* carriers and non-carriers, such as the number of subjects in each group, which may limit the interpretability of their results and lead to weaker conclusions (Barber et al., 2000).

White matter changes are also found in subjects with PD-related cognitive impairment and DLB (Joki et al., 2018; Yang et al., 2023). Commonly seen are White Matter Hyperintensities (WMH), which are white matter lesions analyzed as bright regions in T2-weighted, fluid attenuated inversion recovery (FLAIR) and proton density-weighted MRI images, while they appear darker in T1-weighted MRI scans (Wardlaw et al., 2015; Yang et al., 2023). A meta-analysis identified that enhanced WMH volumes are more prevalent in PDD subjects when compared to those with PD-MCI and PD-NC, indicating it may be a structural marker for PDD (Liu et al., 2021). Previous studies also observed that more WMH volumes in PD and DLB patients predict greater cognitive impairments, especially regarding memory function and processing speed (Dadar et al., 2018; Linortner et al., 2020; Ferreira et al., 2021). *APOE4* has also been associated with increased WMH volumes in PD and DLB subjects in some studies, suggesting it may also contribute to white matter abnormalities known to be linked to cognitive impairment in these conditions (Kandel et al., 2016; Saeed et al., 2018). For example, one study found that PD *APOE4* carriers showed significantly higher WMH volumes when compared to AD *APOE4* carriers (Kandel et al., 2016). This may indicate that PD *APOE4* carriers show pronounced white matter changes, which could potentially contribute to an increase risk of cognitive impairment. However, interpretations must be made with caution, as it is based from a single study. For DLB, Mirza et al. (2019) found their pooled cohort of AD and DLB subjects with *APOE4* to have higher levels of WMH volumes that were linked to worsened learning, language processing, memory, and executive functioning (Mirza et al., 2019). Yet, due to sample imbalances in the cohort (239 AD vs. 50 DLB subjects), interpreting if these observed relationships occurs solely in DLB subjects may be challenging (Mirza et al., 2019). In addition, other studies also did not find an association between *APOE4* and WMH volumes in DLB instead, further complicating conclusions. Ferreira et al. (2023) observed higher WMH volumes in DLB patients without *APOE4*, indicating a lack of association between *APOE4* and increased WMH volumes in DLB subjects (Ferreira et al., 2023). However, since the study focused on those with mild to moderate DLB, the influence of *APOE4* on enhanced WMH volumes could be more apparent in individuals with severe DLB who were ineligible for their investigations (Ferreira et al., 2023). Barber et al. (1999) also found no association between the presence of *APOE4* and white matter lesions including WMH in their *APOE4* cohort that had DLB subjects (Barber et al., 1999). But, the allele frequency of *APOE4* in their DLB cohort was small, specifically 0.30 amongst 22 patients, and should be considered (Barber et al., 1999). Further research is needed to robustly understand the effect of *APOE4* on WMH that may be associated with cognitive impairment in PD and DLB.

Lastly, there is some evidence that changes in ventricular size may be associated with PD-related cognitive impairment. PD-MCI patients showed significantly larger ventricles than those with PD-NC, and that this brain change is associated with cognitive changes including a decline in memory performance (Dalaker et al., 2011). However, two studies found no significant association between enlarged ventricles in their pooled sample of *APOE4* carriers that included those with PD-NC and PD-MCI (Apostolova et al., 2012; Beyer et al., 2013). Therefore, we theorize that the effect of *APOE4* toward PD-related cognitive impairment may not involve ventricle enlargement.

### 3.3 *APOE4* and functional brain changes in Parkinsonian subjects

The relationship between *APOE4* and functional brain measures have been examined in the brains of PD and DLB subjects with *APOE4* in various studies, as detailed in Table 3. However, most reviewed studies involved PD subjects, where there are limited studies in DLB subjects exploring these relationships.

**Table 3:** Details of studies evaluating the functional brain changes including parkinsonian subjects (PD and DLB) with *APOE4* genotype.

Reference	Neuroimaging method	Functional brain changes analyzed	Clinical population	N of *APOE4+*	N of APOE4−	Sex (M/F)	Summary
Nombela et al. (2014)	BOLD sensitive T_2_-weighted MRI	Functional Brain Activity	168 PD for ICICLE-PD study	Site 1: 5	–	Site 1: 25/24	Lower activation of right/left hippocampus, right inferior frontal gyri par triangularis, left inferior frontal gyrus, left occipital and temporo-parieto-occipital areas during Memory Encoding Task in PD *APOE4* carriers than in non-carriers
				Site 2: 5		Site 2: 57/45	
			85 HCs	–	–	Site 1: 27/22	
						Site 2: 17/18	
Shang et al. (2020)	T_1_-weighted Resting State MRI	Functional Connectivity	95 PD-MCI	28	21 *APOE2*, 46 *APOE3*	9/12 *APOE2*, 26/20 *APOE3*, 17/11 *APOE4*	Greater decrease in FC values between bilateral caudate, right middle occipital gyrus, and right middle temporal gyrus in PD-MCI group when compared to HCs FC value for the 3 brain regions significantly lower in *APOE4* carriers than non-carriers within PD-MCI group FC value positively associated with semantic fluency scores in MoCA for only *APOE4* carriers within PD-MCI group
			99 HC	26	31 *APOE2*, 42 *APOE3*	19/12 *APOE2*, 19/23 *APOE3*, 11/15 *APOE4*	
Vijayaraghavan et al. (2014)	[18F]-FDG PET	Glucose Metabolism	9 DLB	6	3	7/2	*APOE4* carriers showed a non-significant trend of lower glucose metabolism in the lateral temporal and the posterior cingulate cortices, but not in frontal cortex when compared to non-carriers CSF APOE protein levels was twofold higher in *APOE4* carriers than in noncarriers; DLB patients had the highest CSF APOE proteins with PDD being the second-highest CSF APOE protein levels negatively correlated with Boston Naming Test and MMSE scores
			10 PDD	3	7	4/6	
			10 PDND	1	8	8/2	
			5 HC	–	5	2/3	
Huertas et al. (2017)	[123I]FP-CIT SPECT	Dopamine Reuptake	298 PD	60	32 *APOE2*, 200 *APOE2−/4*−	60% M	No association between DAT availability analyzed in the caudate nucleus and putamen with *APOE4* status when comparing *APOE4* carriers and non-carriers
Kim et al. (2021b)	DaT-SPECT	Dopamine Reuptake	361 PD	90	271	65 *APOE4*+ males, 173 *APOE4*− males	No significant differences in DAT binding ratios between *APOE4* carriers and non-carriers in PPMI cohort for both the putamen and caudate nucleus Greater decrease in MoCA scores in *APOE4* carriers than non-carriers
Kim et al. (2021a)	DaT-SPECT	Dopamine Reuptake	141 PD (with DaT imaging data)	2 *APOE2/4,* 31 *APOE3*/*4,* 3 *APOE4+/+*	1 *APOE2*/2, 17 *APOE2*/*3,* 87 *APOE3*/*3*	26 *APOE4*+ M, 66 *APOE4*− M	*APOE4* carriers in PPMI cohort had greater cognitive impairment when compared to non-carriers Effect of *APOE4* toward enhanced cognitive function not associated with change in DAT uptake in the caudate nucleus and putamen from baseline (Year 2) to follow-up (Year 4)

APOE4, Apolipoprotein E4; APOE2, Apolipoprotein E2; APOE3, Apolipoprotein E3; APOE4+/+, APOE4 homozygous carriers (two APOE4 alleles); APOE4 +/−, APOE4 heterozygous carriers (one APOE4 allele); APOE4−/−, APOE4 non-carriers (No APOE4 allele); APOE2−/4−, Subjects who do not have APOE2 and APOE4 alleles; DLB, Dementia with Lewy Bodies; HC, Healthy Controls; PD, Parkinson’s Disease; PDND, PD with no Dementia; PDD, PD with Dementia; PD-MCI, PD with Mild Cognitive Impairment; MoCA, Montreal Cognitive Assessment; PPMI, Parkinson’s Progression Markers Initiative; FC, Functional Connectivity; MRI, Magnetic Resonance Imaging; BOLD functional MRI, Blood Oxygenation Level Dependent functional MRI; [18F]-FDG PET, [18F]-fluorodeoxyglucose Positron Emission Tomography; DAT, Dopamine Transporter; DAT-SPECT, Dopamine Transporter-Single-Photon Emission Computed Tomography; [123I]FP-CIT SPECT, I-N-ω-fluoropropyl-2β-carbomethoxy-3β-(4-iodophenyl) nortropane Single-Photon Emission Computed Tomography; ICICLE-PD, The Incidence of Cognitive Impairment in Cohorts with Longitudinal Evaluation-Parkinson’s Disease Study; M, males; F, females.

Functional abnormalities as measured using functional MRI (fMRI), a non-invasive technique that quantifies neural activity by assessing blood oxygen levels, have been found to be relevant in subjects with PD-related cognitive impairment (Sasikumar and Strafella, 2020; Yen et al., 2023). This includes changes in functional activity in specific brain regions, such as reductions in the prefrontal cortex, caudate nucleus, and fronto-striatal regions when doing cognitive tasks (Sasikumar and Strafella, 2020). It also includes altered functional connectivity as measured using resting fMRI, which quantifies the communication between brain regions through distinct patterns of brain activity amongst them (van den Heuvel and Hulshoff Pol, 2010; Baggio and Junqué, 2019). For example, van Eimeren et al. (2009) determined disruptions in the functional connectivity of the Default Mode Network when investigating PD patients, a network crucial for executive function and greatly defective in those experiencing PD-related cognitive impairment (van Eimeren et al., 2009). Limited fMRI evidence is available on the relation between *APOE4* and functional activity as well as connectivity in the brains of those with PD. Of the studies reviewed, reductions in functional activity and connectivity were overall found within specific subcortical regions, including the caudate and hippocampus, as well as posterior cortical regions, which was then associated with cognitive changes. In particular, Nombela et al. (2014) reported significant reductions of activity during a memory encoding task in 10 subjects with early PD and *APOE4* when compared to *APOE2* and *APOE3* carriers (Nombela et al., 2014). These reductions were observed in their right and left hippocampus, and posterior cortical areas, specifically the right inferior frontal gyri pars triangularis, left inferior frontal gyrus, left occipital, and temporo-parieto-occipital areas (Nombela et al., 2014). However, Nombela et al. (2014) acknowledged that the sample size for their *APOE4* carriers is small, which is inferred to potentially lead to biased findings (Nombela et al., 2014). To correspond, Shang et al. (2020) examined in 28 Chinese individuals with PD-MCI and *APOE4* that significantly lower functional connectivity values between the bilateral caudate nucleus and posterior cortical regions, including the right superior occipital and middle temporal gyrus, was significantly associated with reduced semantic fluency scores when compared to PD-MCI *APOE2* and *APOE3* carriers (Shang et al., 2020). Notably, the cognitive domains analyzed in both studies have been previous analyzed to be associated with those with PD-related cognitive impairment. Memory encoding has been evaluated to be adversely impacted in PD-MCI patients when compared to those with normal cognition (Weintraub et al., 2004; Gratwicke et al., 2015; Devignes et al., 2021) Worsened semantic fluency predicts progression to PDD, and is more disrupted in those with PD-MCI than those with PD-NC (Bohnen et al., 2011; Ye et al., 2017; Yang et al., 2022). Thus, both studies suggest that *APOE4* may play a role in PD-related cognitive impairment by affecting the activity of and communication between specific brain regions essential for cognitive processes deficient in PD, especially posterior cortical areas.

Changes in glucose metabolism as measured by [18F]-Fluodeoxyglucose ([18F]-FDG) is also prevalent in PD subjects with cognitive impairment. Both PDD and PD-MCI subjects exhibit distinct brain patterns of glucose hypometabolism associated with changes in memory, attention, executive function, and visuospatial processing (Garcia-Garcia et al., 2012; Meyer et al., 2017). However, only one study exhibited the relationship between *APOE4*, cognitive status, and altered glucose metabolism in those with PD and DLB (Vijayaraghavan et al., 2014). Vijayaraghavan et al. (2014) observed a trend of reduced glucose metabolism in the posterior cingulate and lateral temporal cortex in their combined cohort of *APOE4* carriers (PD-NC, PDD, and DLB subjects) when compared to non-carriers (Vijayaraghavan et al., 2014). Interestingly, CSF APOE protein levels were also found to be significantly higher in their DLB and PDD *APOE4* carriers compared to non-carriers, and that these levels were negatively correlated with glucose metabolism in the lateral temporal cortex and posterior cingulate, as well as with cognitive scores on the Boston Naming Test and MMSE. These results are consistent with previous literature that examined the prevalence of reduced glucose metabolism in these regions in PD patients who converted to dementia, and in PD-MCI and PDD patients when compared to cognitively unimpaired individuals (Vander Borght et al., 1997; Bohnen et al., 2011). In addition, the posterior cingulate is important for attention, and the lateral temporal cortex is essential for language comprehension, facial recognition, and visual processing, all of which are adversely affected in PD individuals with cognitive impairment (Vander Borght et al., 1997; Watson and Leverenz, 2010; Goldstein et al., 2017; Vogt, 2019; Fang et al., 2020; Vijayaraghavan et al., 2014). Thus, we infer that reduced glucose metabolism in specific brain areas important for cognitive function in PD and DLB is linked to *APOE4*, leading to adverse cognitive changes. However, such conclusions are based on limited evidence, and further research is warranted to explore these associations more comprehensively.

Lastly, reduced dopamine neurotransmission has been a key characteristic of PD, occurring from the degeneration of dopaminergic neurons in the substantia nigra and midbrain (Triarhou, 2013). Though its effects are associated with its motor features, some studies report that it may also be linked to cognitive impairment in PD patients (Triarhou, 2013; Fang et al., 2020). Dopamine-Transporter (DAT)-Single Photon Emission Tomography (SPECT) imaging measures dopamine neurotransmission by visualizing the quantity of DAT, a protein found in the endings of presynaptic dopaminergic neurons that facilitate dopamine reuptake from synapses (Akdemir et al., 2021). Specific to *APOE4* and PD, two studies found no significant differences in DAT binding between patients with and without polymorphism in the caudate nucleus and putamen using DaT-SPECT (Huertas et al., 2017; Kim et al., 2021b). Similarly, Kim et al. (2021a) observed that individuals with *APOE4* experience greater cognitive impairment than those without it. Yet, this relationship was not linked with significant reductions in DAT uptake in the caudate and putamen as well between baseline and follow-up (Kim et al., 2021a). Thus, we infer that *APOE4*’s impact toward PD-related cognitive impairment may not include dopaminergic processes.

## 4 Discussion

This review utilized neuroimaging evidence from multiple human studies to assess the potential impact of *APOE4* on brain changes in individuals with Parkinsonisms and cognitive status. It is recognizable that specific brain regions with an essential role in cognitive function consistently showed structural, functional, and/or amyloid-beta protein pathologies in PD individuals with *APOE4* in some studies identified. As discussed and outlined in the tables provided, this includes posterior cortical regions, including parietal, occipital, and temporal cortices; frontal regions, including the frontal and prefrontal cortex; and subcortical regions such as the anterior and posterior cingulate, precuneus, caudate nucleus, and middle temporal gyrus (Maetzler et al., 2009; Gomperts et al., 2012; Nombela et al., 2014; Vijayaraghavan et al., 2014; Akhtar et al., 2017; Rane et al., 2018; Shang et al., 2020; Jung et al., 2021). Specific brain regions crucial for cognition also showed more significant structural and functional alterations in DLB *APOE4* carriers between some of the studies reviewed (Maetzler et al., 2008; Gomperts et al., 2012; Vijayaraghavan et al., 2014; Ferreira et al., 2020; Jung et al., 2021; Diaz-Galvan et al., 2023). Given this, *APOE4* may play a role in the development and progression of PD- and DLB-related cognitive impairment by influencing adverse changes in particular brain areas known to contribute to cognitive function.

Notably, *APOE4* has been found in some studies to be associated with increased amyloid-beta proteins in the brains of PD patients with cognitive impairment and DLB. This strengthens the idea that *APOE4* may play a role in contributing to AD-like pathology in these Parkinsonisms. Thus, it is inferred that its role on amyloid-dependent mechanisms in AD pathogenesis to facilitate enhance amyloid-beta protein pathology may also be similar in subjects with PD-related cognitive impairment and DLB, considering the commonalities between AD, PDD, and DLB, as previously discussed. We note however that no imaging studies were found that examined the relationship between *APOE4* and tau in those with PD, PD-related cognitive impairment, and DLB. Over the years, various PET radiotracers have been developed to image tau proteins (Groot et al., 2022; Mena and Strafella, 2022). Yet, it is possible that no tau imaging studies were found due to the known challenges for tau radiotracers, including off-target binding, and difficulties of radiotracers binding to tau aggregates since they form intracellularly (Marquié et al., 2017; Groot et al., 2022; Mena and Strafella, 2022). Due to this gap, it may be worthwhile in the future to analyze *APOE4*’s impact on tau pathology in PD-related cognitive impairment and DLB using neuroimaging, given the prevalence of tau aggregation in these disorders, and *APOE4*’s known association with tau in AD.

Furthermore, the mechanisms by which *APOE4* specifically contributes to the structural and functional changes observed in the reviewed studies remains poorly understood in individuals with PD and DLB. However, considering that such changes have also been found in diagnosed and at-risk AD patients with *APOE4* (Drzezga et al., 2005; Lind et al., 2006; Trivedi et al., 2006; Xu et al., 2009; Liu et al., 2010; Spampinato et al., 2011; Canuet et al., 2012; Ossenkoppele et al., 2013; Wesnes et al., 2014; Wang et al., 2015; Mirza et al., 2019; Abushakra et al., 2020), it is plausible to hypothesize that *APOE4* contributes to these brain pathologies in PD and DLB through mechanisms implicated in AD pathogenesis, overall leading to cognitive impairment.

For instance, the association between gray matter changes, including cortical thickness and GMV, with *APOE4* in PD and DLB may be attributed to *APOE4’s* impact on impaired cholesterol transport, given cholesterol’s critical role in maintaining neuronal health and synaptogenesis, as previously outlined. However, this association may also be driven by *APOE4*’s contributions toward mechanisms discussed in AD research known to be associated with neurotoxicity, neuronal cell death and synaptic loss, including enhanced amyloid-beta and tau pathology, neuroinflammation, as well as mitochondrial dysfunction (Goel et al., 2022). In addition to *APOE4’s* effect toward AD-like pathology as outlined earlier, which have been found to be associated with neuronal damage, *APOE4* is linked with heightening neuroinflammation in response to these pathologies, and overall neurodegeneration (Yamazaki et al., 2019; Goel et al., 2022). For example, APOE4 has been found to be associated with driving a pro-inflammatory environment in the brain, such as by enhancing the activation of pro-inflammatory microglia, as well as increasing pro-inflammatory cytokine levels as observed in mouse models (Vitek et al., 2009; Shi et al., 2017; Yamazaki et al., 2019). *APOE4* also affects microglial and astrocytic function when compared to *APOE3*, such as reducing their ability to phagocytose and clear amyloid-beta plaques, respectively (Krasemann et al., 2017; Yamazaki et al., 2019; Parhizkar and Holtzman, 2022). These pathological events contribute to sustained chronic inflammation, exacerbating neuronal cell death and synaptic loss as observed in AD (Krasemann et al., 2017; Yamazaki et al., 2019; Parhizkar and Holtzman, 2022). Correspondingly, *APOE4* has been examined to impair mitochondrial function such as by increasing levels of ROS production, leading to aberrant oxidative stress, as well as disrupting calcium homeostasis by enhancing the influx of calcium ions into mitochondria, all of which contribute to neurotoxicity and neuronal damage (Goel et al., 2022; Mahley, 2023; Pires and Rego, 2023). Therefore, we also infer that *APOE4* contributes to the found gray matter changes in PD and DLB, potentially influencing risk and development to cognitive impairment, through pathological pathways that increase neuroinflammation and impact mitochondrial function as well.

Moreover, the association of higher WMH volumes observed in PD and DLB subjects with *APOE4* may potentially be due to *APOE4’*s role toward vascular function as observed in AD. WMH are often presumed to be vascular in origin, representing cerebrovascular lesions as a result of ischemic changes in the brain (Alber et al., 2019). Notably, *APOE4* has been associated with impairments in cerebrovascular function in AD, such as contributing to reduced cerebral blood flow, vascular density, and disrupting the integrity of the blood brain barrier (Yamazaki et al., 2019). Therefore, there is a possibility that *APOE4* enhances the accumulation of WMH in PD and DLB subjects to facilitate cognitive impairment by contributing to adverse cerebrovascular changes.

Further, the association between *APOE4* and reductions in functional activity and connectivity examined in PD subjects may reflect *APOE4’*s role toward synaptic processes, as also mentioned by Nombela et al. (2014). As observed in AD brains and *APOE4* transgenic mice, *APOE4* has been linked to synaptic degeneration, reductions in dendritic spine density, as well as impaired synaptic transmission (Yamazaki et al., 2019; Fernández-Calle et al., 2022). Thus, it is also possible that synaptic dysfunction linked to *APOE4* may impair neuronal signaling both within and between regions, impacting functional activity and connectivity in PD subjects that may exacerbate cognitive impairment.

Lastly, reduced glucose metabolism found in specific brain regions of *APOE4* subjects with PD and DLB may likely be due to *APOE4’s* association with glucose hypometabolism (Yamazaki et al., 2019). *APOE4* has been found to reduce glucose uptake by downregulating signaling pathways as well as decreasing proteins critical for the regulation of such process as examined in preclinical studies (Keeney et al., 2015; Yamazaki et al., 2019). APOE4 has also been examined to impair insulin signaling and glycolysis when compared to APOE3 as seen in transgenic mice, such as by impairing the trafficking of insulin receptors (Zhao et al., 2017). Thus, it is inferred that *APOE4* may influence decreases in glucose metabolism in those with PD and DLB that may contribute to cognitive impairment by adversely affecting mechanisms that may primarily affect glucose uptake. Overall though, all proposed mechanisms are speculative due to limited exploration within PD and DLB cohorts, with most of the current understanding derived from AD research. It is also possible that *APOE4’s* effect in enhancing alpha-synuclein pathology is associated with the brain changes examined, considering it a major protein pathology in PD and DLB, as well as its role in neurodegeneration independent of AD-like changes (Davis et al., 2020; Zhao et al., 2021; Calabresi et al., 2023). Therefore, more research is needed to validate what APOE4-dependent mechanisms are specifically involved in the brain changes found in those with PD and DLB that may potentially be correlated to adverse changes in cognitive function. This may also provide further insight on whether such mechanisms are similar or distinct to AD.

It should be noted also that some studies provided evidence that *APOE4* may not play a role in PD-related cognitive impairment instead. Negative findings may indicate that *APOE4* is not the sole factor driving cognitive impairment in PD and DLB. For example, differences in cognitive outcomes in Lewy body diseases (including PD, PDD and DLB), such as timing, severity and pace of decline, have been discussed to not only be explained by the effects of *APOE4*, but also by other individual factors (Carceles-Cordon et al., 2023). This includes the impact of other genes, such as *GBA, SNCA,* and *BIN1*, and environmental factors, such as traumatic injuries and pesticide exposure (Carceles-Cordon et al., 2023). Thus, this indicates that PD-related cognitive impairment and DLB cannot be fully explained by *APOE* status alone. In addition, factors such as family history, sex, smoking status, and cholesterol levels are discussed to explain why some *APOE4* carriers do not develop AD, and why not all AD subjects carry the allele (Pires and Rego, 2023). Thus, the role of *APOE4* in PD and DLB related cognitive impairment is likely complex, where a combination of environmental and genetic factors contribute to cognitive impairment. Further research is needed to clarify if *APOE4* directly influences cognitive impairment in PD and DLB, or whether other factors interact with *APOE4* to contribute to cognitive changes.

**Figure 2:**
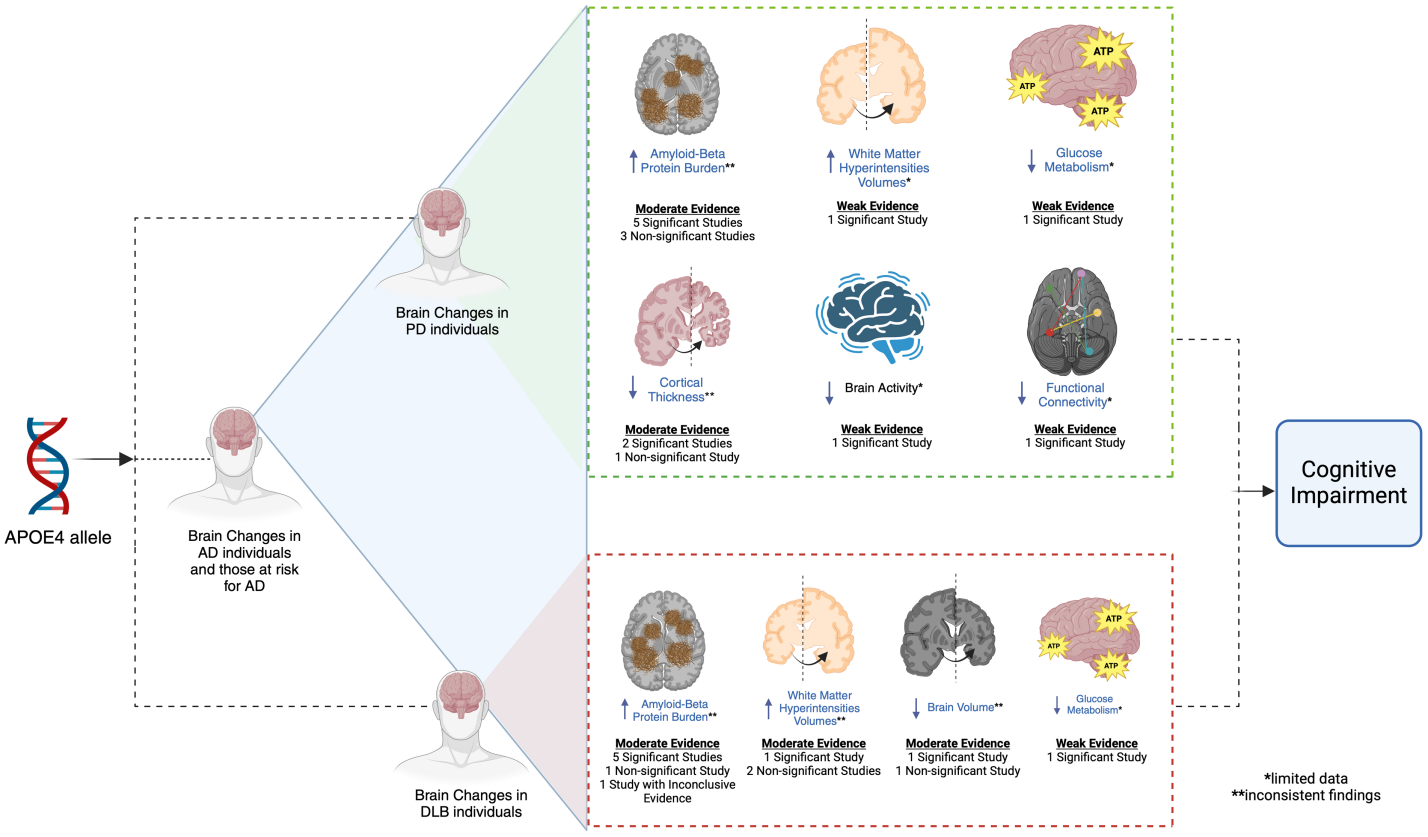
Influences of *APOE4* on brain changes in PD and DLB that may be associated with cognitive impairment. *APOE4* has been found to be associated with brain changes in PD and DLB. These brain changes in PD and DLB *APOE4* carriers have also been observed in diagnosed and at-risk AD subjects carrying the *APOE4* allele. In PD, *APOE4* may play a role toward cognitive impairment by influencing enhanced amyloid-beta protein burden and reduced cortical thickness in brain regions important for cognitive function, similarly found in the brains of those with AD. To note however, there are inconsistent findings within the literature, as other studies found no effect of *APOE4* toward such brain changes. *APOE4* may also be associated with increased WMH volumes, as well as decreased glucose metabolism, brain activity, and functional connectivity in those with PD, potentially leading to cognitive impairment, just like in AD as well. Yet, such inferences are based on limited data, with only one study to make conclusions. In DLB, *APOE4* may play a role in cognitive impairment by influencing enhanced WMH volumes and amyloid-beta protein pathology, as well as reduced brain volume and glucose metabolism in regions essential for cognition also. However, there are limited as well as inconsistent results in the literature, with some studies reporting no association of *APOE4* toward these brain changes in DLB patients. To evaluate the strength of the evidence, findings of *APOE4*’s effect toward the brain changes were based on consistency and the number of studies found. Brain changes with inconsistent findings, where studies reported both significant and non-significant associations with *APOE4*, were categorized as having “moderate” evidence for *APOE4’s* influence on brain changes that may lead to cognitive impairment in PD or DLB. Brain changes associated with *APOE4* but only supported with a single study were classified as having “weak” evidence due to the limited data available. One study was deemed to provide “inconclusive” evidence toward the effect of *APOE4* toward amyloid-beta burden in DLB, as they found significant differences in amyloid-beta during baseline but not during follow-up when comparing DLB *APOE4* carriers and non-carriers.

There are several limitations when making conclusions regarding the articles reviewed. Numerous studies have small sample sizes, which may challenge the ability to draw accurate and reliable conclusions. Future research should attempt to use larger sample sizes to better assess the association between brain changes with *APOE4* in PD and DLB that may be linked to cognitive impairment. In addition, the limited availability of neuroimaging findings investigating the association of *APOE4* and brain changes in PD restricts a robust understanding on how such relationship can be linked to PD-related cognitive impairment (See Figure 2). This is evident in studies analyzing changes in glucose metabolism, brain volume and activity, functional connectivity, and WMH volumes in PD *APOE4* carriers, where only one study was used to evaluate potential relationships between *APOE4* status, brain alterations, and cognitive function. Thus, more neuroimaging studies are needed to advance knowledge on the impact of *APOE4* toward the brain that may facilitate PD-related cognitive impairment. Inconsistent results between studies are also noted, especially regarding the possible effect of *APOE4* toward amyloid-beta burden for both PD and DLB, reduced cortical thickness in PD patients, and alterations in brain volumes and WMH in DLB patients (See Figure 2). Therefore, more studies are needed to clarify these conflicting findings and strengthen interpretations of *APOE4*’s effects on cognitive changes in PD and DLB.

Nevertheless, identifying the potential role of *APOE4* toward the brain that may influence cognitive impairment in parkinsonian individuals can provide crucial insight on the importance of genetic factors as clinical biomarkers for this non-motor symptom. This may aid early detection of those susceptible to cognitive impairment in PD and DLB patients, overall facilitating personalized treatments to better improve patient outcomes.
